# A Note on Its Treatment and Prevalence

**Published:** 1921-09-03

**Authors:** 


### DIABETES.

### A Note on its Treatment and Prevalence.

Since the war a number of medical writers have recorded an increasing prevalence and mortality from diabetes, and although it is difficult to generalise in connection with a condition of such uncertain ætiology, yet a number of facts certainly suggest this conclusion. In Holland, for example, during the rationing period, official figures at the food offices prove that there existed some 11,000 recognised diabetics in the country—*i.e.*, one to every 600 of population, and a decided increase upon any previously recorded figures. Correspondingly, during the period of food shortage it has been authoritatively claimed that there was a decided check to this tendency; an experience which is closely paralleled by the claim that the number of diabetics could be tremendously reduced by a general prevention of obesity. But the food shortage having largely disappeared, the number of diabetics is now rising again. Actually, it might be more accurate to claim as the direct cause the increased consumption of sugar, and it is noteworthy that in America, for example, the average consumption of sugar per individual has already risen to nearly 2 lb. per week. Also, in the *Indian Medical Gazette*, McCay and others have recorded some interesting observations upon diabetes among the Hindus. Essentially, they find that the hard-working Mohammedan, with a meat diet, escapes, whereas the Hindu living in exactly similar surroundings and climate is remarkably prone to diabetes. The obvious, and probably the true, explanation lies with the latter's prolonged carbohydrate dietary. The disease produced is a true diabetes from the first, but mild in nature, and is amenable to the simpler forms of treatment. A sugar-free urine can be obtained in a few weeks by a low carbohydrate diet, and thereafter " re-educative " methods of feeding are employed with considerable success. Admittedly the illustration will not cover all the cases and types met in European practice, but the direct influence of a heavy diet, with a proportionate excess of carbohydrate, in starting and maintaining a *true* diabetes (as compared with a simple glycosuria) seems definitely established. The known tendency towards a carbohydrate dietary, particularly in the cities, and the suggested increase in diabetes may well be viewed as cause and effect.

Any general figures at present available include, however, many cases of sugar appearing in the urine which cannot possibly be thus classified and explained. It is interesting, therefore, to note some recent observations upon the exceptions. Lately Langfeldt gave an elaborate account of two years' research work upon dogs from which a portion of the pancreas had been removed, and his results all confirm the predominant *rôle* played by this organ in the development of diabetic phenomena. In another case sugar was injected directly into the circulation of depancreatised dogs, it being definitely shown that the substance could not be metabolised effectually by these animals. On the other hand, a large number of *post-mortem* records upon known diabetic patients go to prove that in a high percentage of cases nothing whatever is found to be wrong with the pancreas. Therefore, we must be careful how we lay blame upon this gland. The appearance of true glycosuria appears, upon present evidence at least, to depend upon a *balance* of endocrine or internal glandular secretions, and diabetes mellitus may result from any tendency to tip the balance towards diabetes in a person whose metabolism is wavering.

In attempts to define and control this balance considerable attention has been devoted to the so-called glucose " excretion threshold," which may be described as that percentage of sugar in the blood at which glucose begins to appear in the urine. A certain number of definite points have been established, but these are interesting only to the expert, for, disappointingly enough, it is found that concentration of sugar in the blood cannot be said to account for more than a few of the diabetic cases. There are undoubtedly other factors concerned. It is possible to raise or lower the threshold by experimental injections of certain glandular extracts, which is strongly suggestive of an endocrine balance, as already described.

As a simpler type, however, one might take the now well-recognised " Renal " glycosuria. It is not yet proved whether this condition is so simple that it is accounted for by a mere increased permeability of the kidney to sugar, but the evidence tends in that direction. In any case, the amount of sugar passed depends directly upon the dietetic, especially carbohydrate, excess, and many of our ordinary cases amount to this and nothing more. Certainly they show none of the constitutional symptoms of diabetes, and frequently provide us with instances of transient or recurrent glycosuria. Fortunately, their control and treatment are relatively simple, and only from the point of view of life insurance do they present difficulty to the practitioner. Even then a very satisfactory tolerance test can be carried out as follows (Williamson): 100 grams of glucose, dissolved in ¼-litre of water or tea, are given upon an empty stomach before breakfast. The urine may be tested at three-quarters hour and two hours afterwards. The perfectly healthy person, in whom the presence of sugar at the original examination was a mere accident due to dietetic excess, will show no glycosuria—after 100 grams of glucose—and may certainly be accepted by the company. The finding of glucose after this test is, however, held to show defective metabolism or impaired renal function.

With regard to treatment of what are vaguely termed true diabetics, one finds that diet-restriction methods are still unreplaced. The plan of enforcing a rigid fast has met with much adverse criticism. Allen and many others suggest, in recent notes, that in most cases life, strength, and assimilation can be preserved for a much longer period by a degree of *under-nutrition* suited to the severity of the diabetes and accompanied by limitation of fat in the diet. In severe cases, of course, to free the urine from sugar so great a reduction of food must be made that death rapidly ensues. Therefore there seems little doubt that the best policy to pursue is summed up in the words, a restricted diet graded to the individual case, allowing, if necessary, a moderate glucosuria to remain.

One or two points come to mind concerning this gradation of diet. Cammidge has provided a useful suggestion for arriving at a final routine after observations on the amount of carbohydrate that may be allowed to the individual patient. He recommends that the patient's tolerance be taken as about 20 per cent. below that amount of carbohydrate which is just enough to produce a trace of sugar in the urine. He also gives a good method of preparing sugar-free vegetables. When carrots, beetroot, or artichokes are boiled for a quarter of an hour, and the process repeated after the water has been removed, the vegetables have had all the sugar removed. The same result can be achieved by extracting them in a shredded state at 60° C. for an hour, the water being frequently changed.

